# A multimodal deep learning system to distinguish late stages of AMD and to compare expert vs. AI ocular biomarkers

**DOI:** 10.1038/s41598-022-06273-w

**Published:** 2022-02-16

**Authors:** Kaveri A. Thakoor, Jiaang Yao, Darius Bordbar, Omar Moussa, Weijie Lin, Paul Sajda, Royce W. S. Chen

**Affiliations:** 1 Department of Biomedical Engineering, Columbia University, New York, 10027 USA; 2 Department of Electrical Engineering, Columbia University, New York, 10027 USA; 3 Department of Ophthalmology, Edward S. Harkness Eye Institute, Columbia University Irving Medical Center, New York, 10032 USA; 4 Department of Radiology (Physics), Columbia University, New York, 10027 USA

**Keywords:** Diagnostic markers, Biomedical engineering, Translational research

## Abstract

Within the next 1.5 decades, 1 in 7 U.S. adults is anticipated to suffer from age-related macular degeneration (AMD), a degenerative retinal disease which leads to blindness if untreated. Optical coherence tomography angiography (OCTA) has become a prime technique for AMD diagnosis, specifically for late-stage neovascular (NV) AMD. Such technologies generate massive amounts of data, challenging to parse by experts alone, transforming artificial intelligence into a valuable partner. We describe a deep learning (DL) approach which achieves multi-class detection of non-AMD vs. non-neovascular (NNV) AMD vs. NV AMD from a combination of OCTA, OCT structure, 2D b-scan flow images, and high definition (HD) 5-line b-scan cubes; DL also detects ocular biomarkers indicative of AMD risk. Multimodal data were used as input to 2D-3D Convolutional Neural Networks (CNNs). Both for CNNs and experts, choroidal neovascularization and geographic atrophy were found to be important biomarkers for AMD. CNNs predict biomarkers with accuracy up to 90.2% (positive-predictive-value up to 75.8%). Just as experts rely on multimodal data to diagnose AMD, CNNs also performed best when trained on multiple inputs combined. Detection of AMD and its biomarkers from OCTA data via CNNs has tremendous potential to expedite screening of early and late-stage AMD patients.

## Introduction

Age-related macular degeneration (AMD) is a leading cause of blindness, impacting millions of people worldwide^1,2^. It is estimated that more than 14% of middle and older-aged adults (43–86 years of age) will have AMD within the next 15 years^3^. Choroidal neovascularization (CNV) is a key feature/biomarker of progression to neovascular AMD (NV AMD), a late stage of AMD which can rapidly lead to blindness if left untreated^4^. Therapy targeting vascular endothelial growth factor (anti-VEGF) has enabled significant improvement in management of NV AMD in recent years^5^. To identify patients most likely to benefit from anti-VEGF treatment, optical coherence tomography (OCT) and optical coherence tomography angiography (OCTA) have become critical noninvasive tools for qualitative and quantitative assessment of features (such as retinal thickness and intra/subretinal fluid) both to measure disease progression and response to treatment^6^. However, these imaging modalities generate a tremendous amount of volumetric data for each patient, with each volume potentially containing critical evidence for determining disease state. To expedite clinician workflow and expand the amount of data a single ophthalmologist can effectively parse, artificial intelligence (AI) may be able to serve as a ‘team-mate’ by screening data volumes that unambiguously belong to specific disease classes. Past applications of machine learning/computer vision (ML/CV) approaches to retinal fundus images and fluorescein angiography image sequences, using pyramids of histograms of oriented gradients (PHOG) and Adaboost, achieved multi-class (normal (non-AMD) vs. dry (NNV) vs. wet (NV) AMD) classification accuracies in the low-to-mid 80% range; however, ML/CV approaches applied to OCTA data are less common^7^. Past deep learning approaches toward this task have focused mainly on applying convolutional neural networks (CNNs) to 2D color fundus photographs^8^. A handful of recent studies have shown promise at distinguishing AMD from non-AMD eyes^9^ using features from OCT images. These approaches have enabled distinction of exudative vs. non-exudative AMD^10^, localization of image correlates that suggest utility of anti-VEGF treatment^11^, and retinal-pathology based recommendations for urgent referral^12^. However, there has been less emphasis of deep learning on differentiating between non-AMD and late-stage NNV AMD and NV AMD. Wang and colleagues^13^ achieved detection of CNV vs. no CNV from OCTA cubes, although their work’s emphasis was on segmentation of CNV from OCTA data and hence included pathologies other than AMD. Vaghefi and colleagues^14^ implemented an Inception-Resnet-V2 model to process OCT en-face, OCT-A en-face, and fundus photographs individually as well as in combined form. They observed higher sensitivity and specificity when multiple modalities were combined. However, they used 2D en-face images as inputs in all cases instead of volumes, enabling use of state-of-the-art Inception-Resnet-V2 with few modifications. Also, they distinguished between young healthy, old healthy, and dry AMD patients (non-neovascular AMD patients) rather than differentiating between subtle features of healthy, non-neovascular, and neovascular AMD. We specifically chose not to use state-of-the-art pretrained network architectures in this study, as these models are not amenable to non-2D input data, and many of our inputs are unique 3D cubes (256 × 256 × 5 retinal layers). Hence, we chose to build our own trained-from-scratch models tailored for our unique 3D cube inputs, aiming our scientific question on probing the impact on accuracy of multiple varied input modalities. Jin and colleagues^15^ utilize a novel multimodal feature fusion approach to combine OCT as well as OCTA features via ResNet-50-backbone ‘assistant’ and ‘principle’ pathways as well as ophthalmologist-provided CNV masks to create a unidirectional fusion network which achieves highly accurate CNV activity detection in neovascular AMD patients. While offering a unique multimodal fusion architecture, this method does not address multi-class automated distinction between healthy vs. non-neovascular vs. neovascular AMD, and it also does not handle 3D cube inputs but rather only 2D en-face OCTA inputs. Our work is one of the few to attempt 3-class detection of non-AMD, NNV AMD, and NV AMD, so there are few direct benchmarks for comparison. Inspired by past work^1,16,17^ which used deep learning to detect OCT b-scan biomarkers associated with AMD progression (including intraretinal hyperreflective foci, hyporeflective foci within drusenoid lesions, and subretinal drusenoid deposits, among others), we further quantify performance of our models at detection of five key AMD biomarkers using multiple image inputs. Building on our past work^18,19^, this study offers three key contributions: (1) multi-class detection of non-AMD vs. NNV AMD vs. NV AMD from a multimodal combination of OCTA, OCT structure, 2D b-scan flow images, as well as HD 5-line b-scan cubes, (2) model ablation studies with subsets of the above input modalities to arrive at the input set(s) which optimize multi-class accuracy, and (3) detection of features/biomarkers within these volumes that can be used independently for diagnosis. Concurrently through this second goal, we compare ocular biomarkers detected by humans with those detected by CNNs as a means by which to corroborate/explain the CNN’s decision-making mechanism.

## Methods

### Dataset

**Figure 1:**
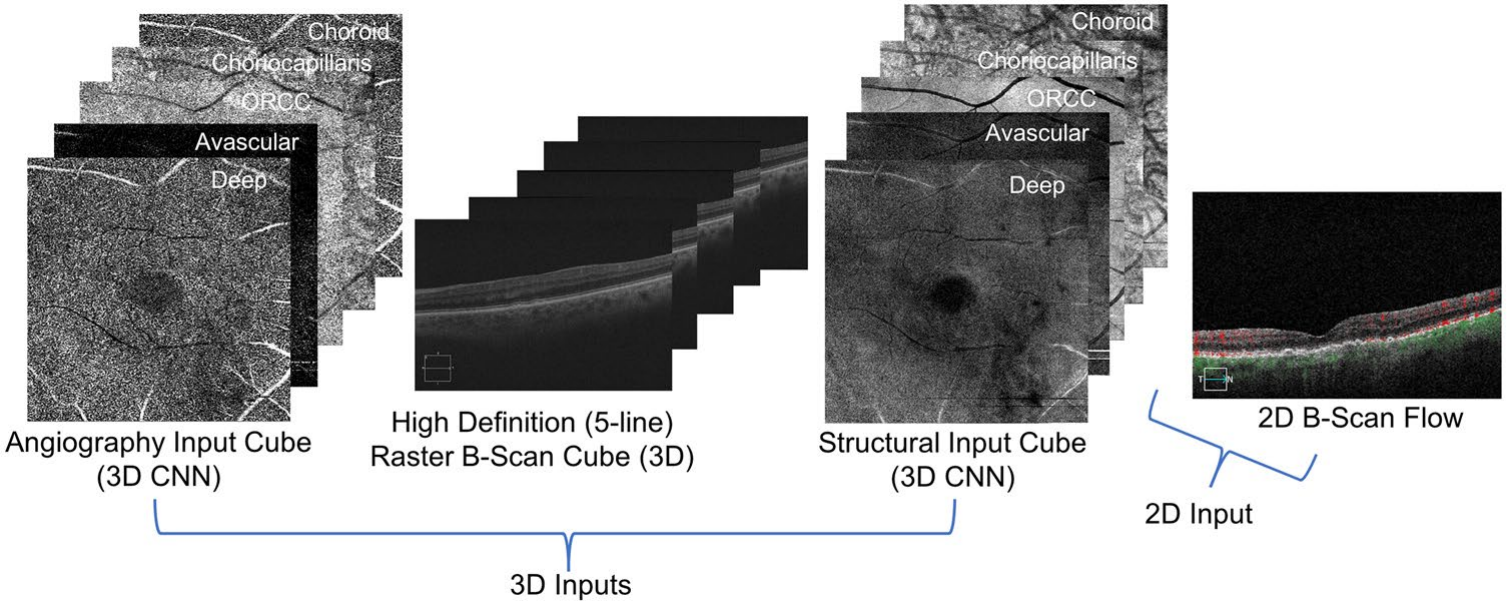
All possible inputs to 3D and 2D CNNs, subsets of which made up inputs to 9 different models. The OCTA and OCT structural cubes for each patient were composed of 5 retinal layers/complexes: deep, avascular, outer retina and choriocapillaris (ORCC), choriocapillaris, and choroid.

OCTA, OCT structure, HD 5-line b-scans, and 2D b-scan flow images from 501 eyes belonging to 305 patients (18 years and older) were obtained retrospectively from Columbia University Medical Center. These eyes were categorized as having no significant vascular pathology (non-AMD), as having non-neovascular AMD (NNV AMD), or as having neovascular AMD (NV AMD) with choroidal neovascularization (CNV). This study (AAAS9395) was approved by the Columbia University Irving Medical Center Institutional Review Board and adheres to the tenets set forth in the Declaration of Helsinki and the Health Insurance Portability and Accountability Act. Written informed consent was obtained from all subjects. The final dataset contains 104 non-AMD eyes, 247 NNV AMD eyes, and 150 NV AMD eyes. Note, this split was enforced at the eye level rather than at the patient level, as a majority (62%) of patients had variation in pathology between their left and right eyes. These were split into training (60%), validation (20%), and test (20%) sets (301 training, 100 validation, and 100 test). Various combinations of 3D and 2D inputs were used to train 9 different CNN architectures described in the next section. The OCTA cubes and OCT structural cubes were composed of the following 5 retinal layers or complexes most informative for detecting CNV: deep, avascular, outer retina and choriocapillaris (ORCC), choriocapillaris, and choroid. Figure 1 shows examples of the four modalities used as inputs to the CNNs (including the layers/complexes comprising the OCTA and OCT structural cubes). All data was collected using a Carl Zeiss Cirrus HD-OCT 5000 device.

### Deep learning model development

**Figure 2:**
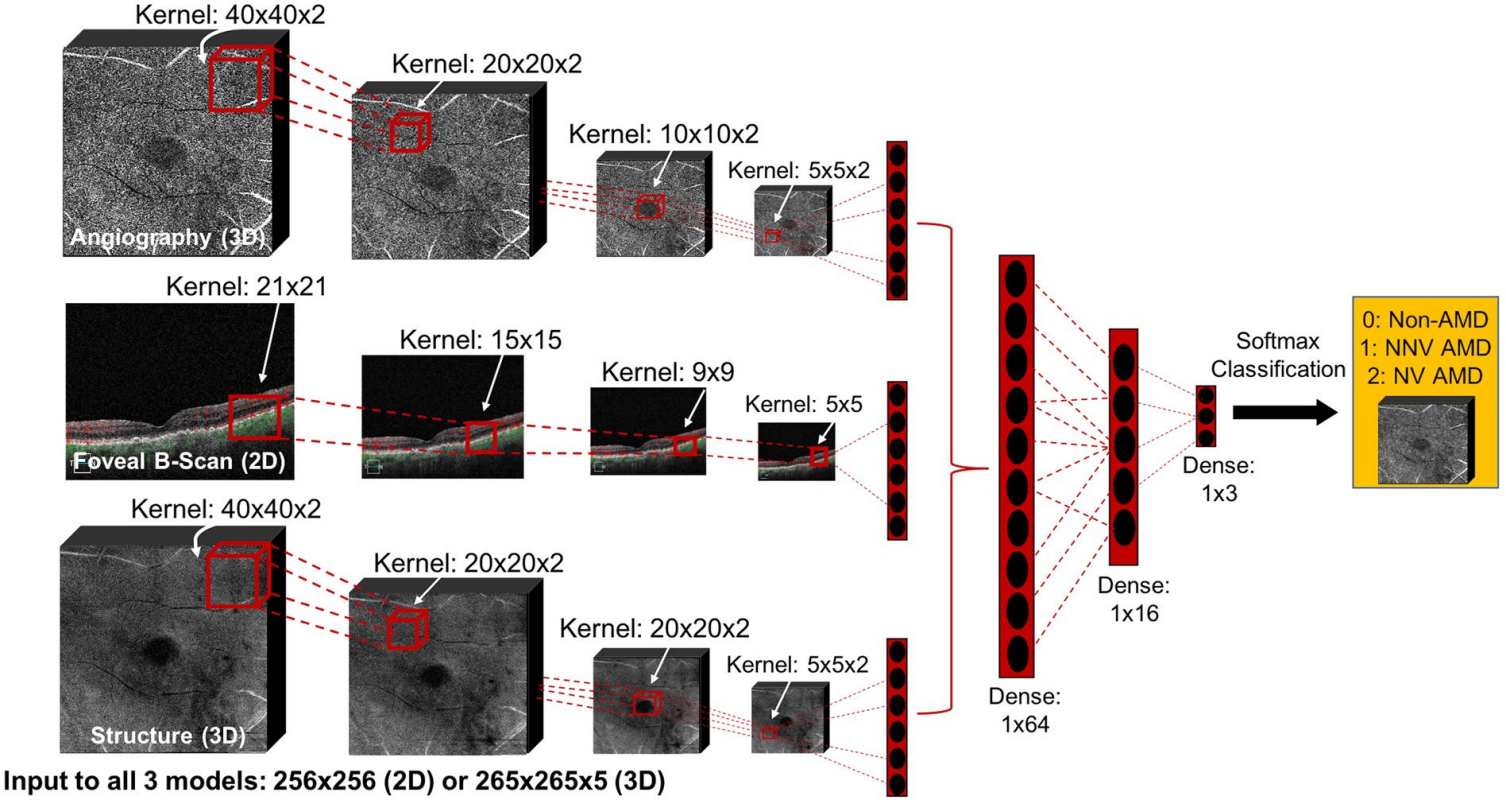
Schematic of OCTA + OCT structure + 2D b-scan flow model [model (3)]. Filter sizes are shown for each modality and layer; features extracted by each modality-specific model are combined via concatenation (long skip connections) to arrive at final multi-class classification.

Hybrid CNNs were constructed and trained respectively on: (1) OCTA, (2) OCTA and OCT structure (en-face structural images generated in OCTA capture), (3) OCTA, OCT structure, and 2D OCT b-scan flow images, (4) OCTA, OCT structure, and HD 5-line OCT b-scan cubes, (5) OCTA, HD 5-line OCT b-scan cubes, and 2D OCT b-scan flow images, (6) OCTA and HD 5-line OCT b-scan cubes only, (7) 2D OCT b-scan flow images only, (8) HD 5-line b-scan cubes only, and (9) OCTA, OCT structure, 2D OCT b-scan flow images, and HD 5-line b-scan cubes combined. The 3D CNN architectures consisted of four convolutional blocks, made up of 5, 8, 10, and 15 filters, respectively (with filter sizes as shown in Fig. 2), followed by Leaky Rectified Linear Unit (LeakyRelu) activation and max pooling with stride of 2 × 2 × 1. To address overfitting, dropout was added after each convolutional layer (increasing in probability from 0.05 to 0.3 with depth), and L1 regularization with regularization strength of 1 × 10^–3^ was used. Different streams of input data were combined via concatenation (a form of long residual/skip connection) after the final dense layer within each modality-specific model. Concatenated features were fed to two dense layers, with 64 and 16 units, respectively, and then were classified via softmax activation into one of three classes (non-AMD, NNV AMD, or NV AMD), as exemplified in Fig. 2 for one of the 9 models (model (3)), which combines OCTA + OCT structure + 2D b-scan flow information. Additional training details are described in past work^18^; dataset imbalance was addressed by enforcing balanced quantities of samples from each of the three classes within the training set (60%) while the validation and test sets were composed of the remaining samples proportionately distributed to achieve a 20%:20% validation-test split without balancing enforced^18^. Figure 2 shows a schematic of how individual modality sub-models were combined: the highlights of our CNN architectures are (1) the ability to ingest multiple 3D input modalities and combine them using feature concatenation via long skip connections and (2) the ability to elucidate the relative contributions of these varied clinical inputs. Early stopping was employed to optimize training, and weights from the best validation epoch were used for testing. All models were trained, validated, and tested on a Lambda Labs Vector server with an RTX 3090 Graphical Processing Unit (https://lambdalabs.com/gpu-workstations/vector).

### Biomarker labeling: assessment via experts and machines

For 482 out of the 501 eyes (19 eyes excluded due to imaging artifacts or presence of pathologies other than AMD), five AMD features/biomarkers were adjudicated by OCTA experts by observing OCTA, OCT structure, 2D b-scans, and HD 5-line b-scans for each patient. The biomarkers evaluated include the following: (1) intra/sub-retinal fluid (IRF/SRF), (2) scar, (3) geographic atrophy (GA), (4) CNV, and (5) pigment epithelial detachment (large PED). IRF expert adjudication was based on the presence of hyporeflective cystoid spaces within the retinal layers on OCT b-scans consistent with fluid within the sensory retina. SRF was based on the presence of hyporeflective space in between the retina and the retinal pigment epithelium (RPE). The presence of CNV was confirmed when signal with a vascular branching pattern was present, typically with a surrounding flow void identified on OCTA as determined by expert judgment. Scar, which forms in later stages of exudative AMD often as a sequela to subretinal hemorrhage, was identified by the presence of a hyperreflective, well-demarcated mass under the retina with disruption of overlying retinal architecture. Geographic atrophy was labeled in areas of RPE atrophy with enhanced OCT signal in regions directly below it. Pigment epithelial detachment can be divided into 3 subtypes: serous PED (filled with optically empty-appearing fluid), fibrovascular PED (filled with heterogeneously hyperreflective material), and mixed PED. If the above criteria were met, respectively for each feature, the feature was labeled as present (1); if not, the feature was labeled as not present/absent (0). These categorical labels were used to train the best-performing models, models (4) and (9), on 482 of 501 eyes (19 eyes were excluded due to imaging artifacts or presence of pathologies other than AMD) to detect binary presence or absence of these features. Five separate CNN feature prediction classification tasks were conducted (one for each feature), with the final prediction of presence or absence arrived at by applying default 0.5 threshold from Sigmoid activation function at the final layer of model (4) or (9), respectively. Five-fold cross-validation was used to obtain feature predictions for one-fifth of the eyes per fold while training on the remaining expert labels; final test accuracy was computed by combining performance across all five folds. (Since we had no prior preference for skewing toward either false positives, FPs, or false negatives, FNs, for any particular feature, we did not explore thresholds other than 0.5; however, if one had prior preference for either FPs or FNs, for example due to known feature prevalence, this threshold could have been further explored.) Both expert labels and CNN feature predictions were compared; for the eyes for which experts and AI diagnoses agreed (341 out of 482), statistical odds ratios were computed using Fisher’s Exact Test^20^ for each biomarker to determine the relative ranking of feature presence as an indicator of disease for both CNNs and human experts. In addition, the accepted clinical feature-based ‘algorithms’ for diagnosing NV AMD and NNV AMD were compared to expert labeling and AI predictions by examining proportions of positive and negative feature labels/predictions for each category of disease, NV AMD and NNV AMD. The clinical ‘algorithm’ for **NV AMD** requires presence of CNV (**CNV+**) and/or presence of IRF/SRF (**IRF/SRF+**) on OCTA; the clinical ‘algorithm’ for **NNV AMD** requires absence of CNV (**CNV−**) and absence of IRF/SRF (**IRF/SRF−**) on OCTA^21^.

## Results

### Model performance

**Figure 3:**
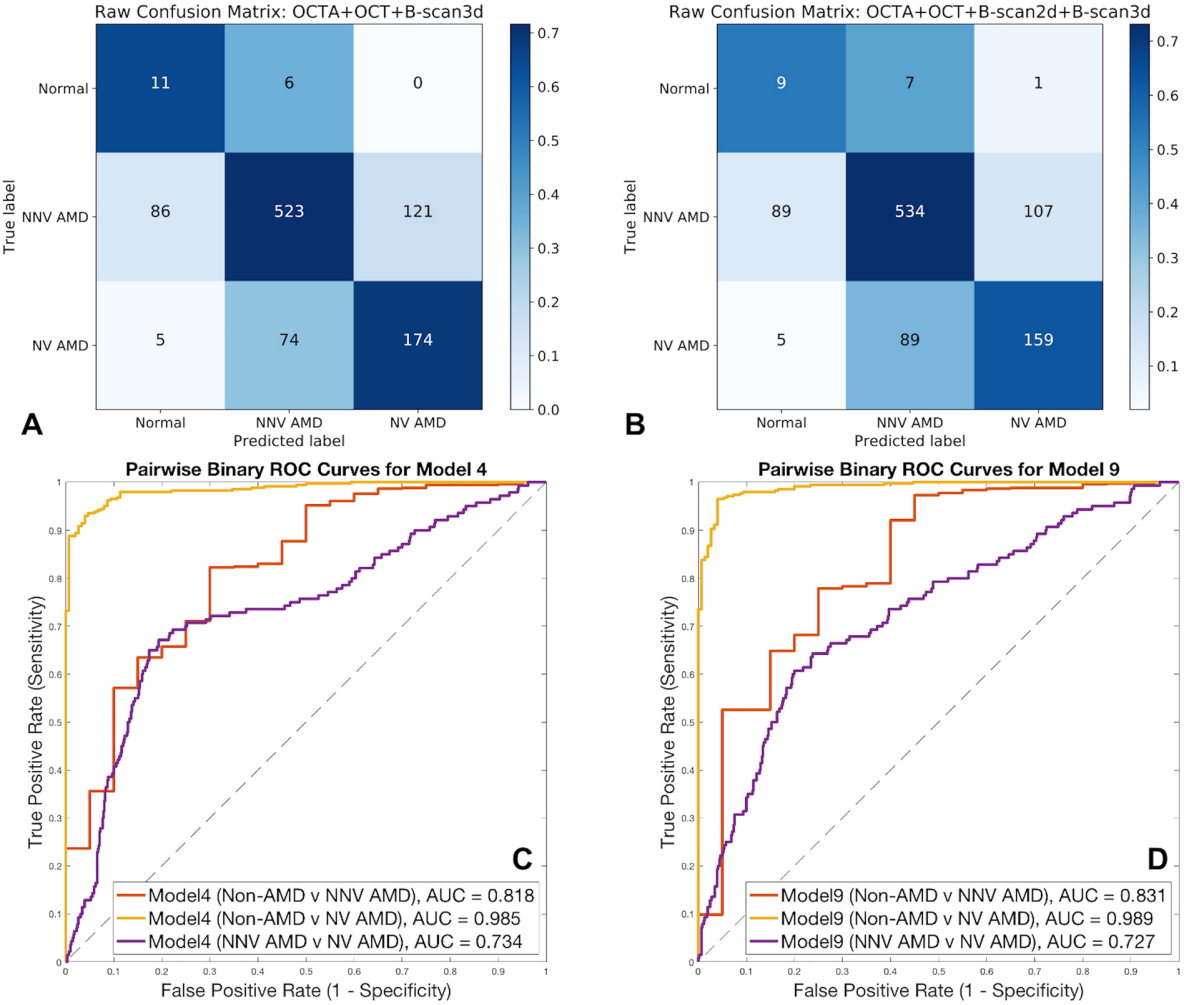
Confusion matrices (**A**) for model (4) and (**B**) for model (9). These are the two top-performing models, with model (4) [left], combining OCTA, OCT structure, and HD 5-line b-scan cubes, slightly outperforming model (9) [right], combining OCTA, OCT structure, HD 5-line b-scan cubes, and 2D b-scan flow images, as can be seen by the higher sum of correct classifications along the diagonal of the confusion matrix at left. Receiver Operating Characteristic (ROC) curves and corresponding Area Under the Curve (AUC) values are shown in (**C**) for model (4) and in (**D**) for model (9). AUC values are slightly higher for model (9) for Non-AMD vs. NNV AMD and for Non-AMD vs. NV AMD classes compared to those of model (4).

Two of the models combining multiple (3 or 4 different) input image modalities exhibited higher overall accuracy compared to single- and dual-input modality models. In particular, model (4) (OCTA cubes + OCT structural cubes + HD 5-line b-scan cubes) and model (9) (OCTA cubes + OCT structural cubes + 2D b-scan flow images + HD 5-line b-scan cubes) achieved highest percent accuracy at multi-class detection of Non-AMD vs. NNV AMD vs. NV AMD. This can be visualized based on the sum of correct classifications on the diagonals of the confusion matrices in Fig. 3A and B, the ROC curves and AUC values in Fig. 3C and D, as well as through the accuracies in Table 1, the precision, recall, F-1 scores in Table 2, and the sensitivities and specificities in Table 3. Confusion matrices and test accuracies for all 9 model architectures are provided in Supplementary Information.

**Table 1:** Mean accuracies and standard errors for all 9 models.

Model Inputs →	(1) OCTA	(2) OCTA + OCT structure	(3) OCTA + OCT structure + 2D B-scan	(4) OCTA + OCT structure + 3D B-scan	(5) OCTA + 3D B-scan + 2D B-scan	(6) OCTA + 3D B-scan	(7) 2D B-scan	(8) 3D B-scan	(9) OCTA + OCT structure + 2D B-scan + 3D B-scan
Training (mean ± standard error)	79.3% ± 1.20%	87.1% ± 1.17%	93.5% ± 1.14%	**94.7** ± **0.97%**	94.4% ± 1.08%	93.4% ± 1.45%	*76.7% ± 1.80%*	*76.6% ± 3.11%*	**95.4%** ± **1.63%**
Validation accuracy	60.4% ± 1.42%	70.5% ± 1.54%	71.0% ± 1.82%	**72.6** ± **1.19%**	64.2% ± 1.40%	65.5% ± 1.65%	*43.3% ± 1.61%*	*44.4% ± 2.26%*	**72.9%** ± **1.72%**
Test accuracy	61.5% ± 1.61%	68.1% ± 1.75%	67.8% ± 1.08%	**70.8** ± **1.12%**	63.7% ± 1.89%	64.8% ± 1.79%	*40.5% ± 1.74%*	*42.4% ± 2.80%*	**70.2%** ± **1.66%**

Each model was run 10 times with a fixed random seed to enable aggregation and comparison across models. Results in bold font are for models with best accuracy; results in italics font are for models with poorest accuracy.

**Table 2:** Precision, recall, and F-1 scores for model (4), composed of OCTA + OCT structure + HD 5-line b-scans (abbreviated as B-scan3d) and for model (9), composed of OCTA + OCT structure + HD 5-line b-scans + 2D b-scan flow inputs (abbreviated as B-scan2d).

Class	Precision (model 4, 9)	Recall (model 4, 9)	F1-score (model 4, 9)
Non-AMD	**0.108**, 0.087	**0.647**, 0.529	**0.185**, 0.149
NNV AMD	**0.867**, 0.848	0.716, **0.732**	0.784, **0.786**
NV AMD	0.590, **0.596**	**0.688**, 0.628	**0.635**, 0.612

In each case, the value for the higher-performing of the two models is in bold.

**Table 3:** Sensitivity and specificity for best-performing models 4 and 9.

Class	Sensitivity (model 4, 9)	Specificity (model 4, 9)
Non-AMD	**0.647**, 0.529	**0.885**, 0.881
NNV AMD	0.716, **0.732**	**0.698**, 0.636
NV AMD	**0.688**, 0.628	0.815, **0.834**

In each case, the value for the higher-performing of the two models is in bold.

### Precision, recall, sensitivity, specificity, F-1 scores, and ROC curves

Table 2 shows the higher recall and F-1 scores for the non-AMD and NV AMD classes for model (4), combining OCTA + OCT structure + HD 5-line b-scan cubes. For the NNV AMD class, model (9) (combining all inputs: OCTA + OCT structure + 2D b-scan flow images + HD 5-line b-scan cubes) slightly out-scores model (4) for recall and F-1 score; model (9) also has slightly higher precision for the NV AMD class. Precision, recall, and F-1 score distributions for both top-performing models are shown in Table 2, and sensitivity and specificity for top models are shown in Table 3. While model (4) exhibits higher sensitivity and specificity than model (9) for the Non-AMD class, model (4) shows lower sensitivity and higher specificity than model (9) for NNV AMD and higher sensitivity and lower specificity than model (9) for NV AMD.

### Human vs. AI feature/biomarker labeling

Results of labeling five AMD biomarkers showed that CNV and GA are important predictors for all (NNV or NV) AMD compared to non-AMD eyes based on Fisher’s Exact Test odds ratios^20^; CNV and GA had odds ratios of 36 and 32 for experts, respectively, and 32 and 26 for the CNN, respectively. Odds ratios for all 5 biomarkers are shown in Table 4 below, with values indicating the ‘odds’ of having any AMD (NNV or NV AMD) or NV AMD only. Using Fisher’s Exact test, we arrived at the odds ratios shown in Table 4, which are consistent in ranking CNV and GA as most indicative of any AMD (NNV AMD and NV AMD, left half of Table 4) compared to non-AMD for humans and machines; for NV AMD, humans and experts both ranked CNV with highest odds ratios of 3904 and 361, respectively (right half of Table 4).

**Table 4:** Odds ratios for experts and for AI for five biomarkers most indicative of NNV or NV AMD are shown in the left half of the table.

All AMD (NV or NNV AMD)			NV AMD only		
Feature	Human expert rank (odds ratio)	Algorithm rank (odds ratio)	Feature	Human expert rank (odds ratio)	Algorithm rank (odds ratio)
CNV	**1 (36)**	**1 (32)**	**CNV**	**1 (3904)**	**1 (361)**
GA	**2 (32)**	**2 (26)**	Scar	2 (109)	3 (65)
Large PED	**3 (18)**	**3 (15)**	IRF/SRF	3 (104)	5 (24)
Scar	**3 (18)**	**3 (15)**	GA	4 (90)	2 (109)
IRF/SRF	**4 (17)**	**4 (7)**	Large PED	5 (62)	4 (56)

For all AMD, both experts and AI agree on feature rank order, with CNV and GA ranked highest. Experts and AI odds ratios for five biomarkers specifically for NV AMD are shown in the right half of the table. For NV AMD, beyond CNV ranked highest, experts and AI show some variation in feature rank order. Features exhibiting agreement in rank between experts and AI are bolded.

In addition to odds ratio analysis to gauge relative importance of these biomarkers for disease risk, we also carried out a comparison between the “clinical algorithm” for detecting NNV and NV AMD using known clinical features and the AI’s feature-level predictions (based on training on expert labeling) to determine if the clinical algorithm was ‘followed’ by the AI. Overall, all five features were predicted with relatively high accuracy (note high chance values due to sparse feature presence) and positive predictive value (PPV) by model (4); IRF/SRF was detected with 85.1% accuracy (chance: 84.0%, 60.0% PPV), scar with 89.0% accuracy (chance: 85.9%, 65.3% PPV), GA with 81.3% accuracy (chance: 76.8%, 62.5% PPV), Large PED with 90.2% accuracy (chance: 86.1%, 66.1% PPV), and CNV with 81.7% accuracy (chance: 71.2%, 75.8% PPV). Of the eyes that were diagnosed (by experts and the AI) with **NV AMD**, all of them (100%) were labeled as having CNV (**CNV+**) by experts; all (100%) that were diagnosed with **NNV AMD** were labeled by experts as not having CNV (**CNV−**). For IRF/SRF, 58% of the patients with **NV AMD** were labeled by experts as **IRF/SRF+**, while 100% of the **NNV AMD** patients were labeled by experts as **IRF/SRF−**. The CNN followed a similar trend: 83% were labeled as **CNV+ **and were diagnosed with **NV AMD**, and 94% were labeled as **CNV−** and were diagnosed with **NNV AMD**; on the other hand, the CNN labeled only 24% of **NV AMD** patients as **IRF/SRF+**, while it labeled 99% of the **NNV AMD** patients as **IRF/SRF−** (results in Table 5). Feature analysis results for model (4) are presented in the main manuscript, while those for model (9) and for 346 of 501 eyes using the best-performing model (2) (without b-scans) from past work^18^ are presented in Supplementary Information (Tables 1S, 2S for model (9) and 3S, 4S, for model (2) for odds ratios and proportions, respectively).

**Table 5:** Proportion of patients diagnosed with NV AMD and NNV AMD (by experts and the AI) labeled for presence (+) or absence (−) of CNV and IRF/SRF biomarkers by the experts and the AI, respectively.

Feature/biomarker and labeler	NV AMD (feature+) (%)	NNV AMD (feature−) (%)
CNV by expert	100	100
CNV by AI	83	94
IRF/SRF by expert	58	100
IRF/SRF by AI	24	99

## Discussion

### Model performance

Just as experts rely on a combination of OCTA and structural b-scan information to diagnose AMD, CNNs also performed best when trained on multiple modalities, including OCTA, OCT structure, and HD 5-line b-scan cubes combined (model (4)), with 3-class distinction accuracy above 70% (with chance accuracy being 33.3%). To put these results into context, the best-performing model, model (4), achieves up to 94.5% ± 1.32% test accuracy, comparable to state-of-the art benchmarks, at binary classification of no CNV (non-AMD) vs. CNV (NV AMD)^13^. Although there are very few existing approaches for achieving multi-class (3-class) detection of non-AMD vs. NNV AMD vs. NV AMD, our accuracy results using multiple 3D input modalities (including OCTA which is relied on by clinicians) are comparable or somewhat higher than the closest state-of-the-art work using cross-validation of 399 ultra-widefield fundus images alone as input to detect these 3 classes (they achieve 73% accuracy for 3-class and 91% accuracy for non-AMD vs. NV-AMD classification, respectively)^22^. The second-place model, model (9), also incorporated 2D b-scan flow images. 2D b-scan flow images may be contributing to reducing performance due to their lower resolution and due to capturing only a single cross-section of the retina. Noteworthy is that these top models outperform models which combine OCTA or OCTA + OCT structural information alone (single and dual modality inputs). Thus, b-scan images, especially HD 5-line b-scan cubes, contribute to improving late-stage AMD detection accuracy compared to OCT structural and/or OCTA information alone even though 2D b-scan flow or HD 5-line b-scan information in isolation do not exhibit high performance. In spite of this encouraging performance, severe overfitting was observed for validation and test data. While increasing dropout in convolutional layers alone did not reduce overfitting sufficiently, increasing regularization strength from 1 × 10^–5^ to 1 × 10^–3^ caused test accuracy to improve slightly. Especially 2D b-scan flow images and HD 5-line b-scan cube inputs in isolation [models (7) and (8)] exhibited poorest 3-class distinction performance, potentially due to overparameterization which could be addressed via use of spatial or depth-wise separable convolutions in future iterations of this work; binary classification performance (CNV vs. Non-AMD) for these two models achieves up to 80.6% ± 2.46% accuracy, interestingly comparable in performance to clinicians using a combination of Spectral-Domain OCT and Fluorescein Angiography to detect Type I CNV^23^. A thorough comparison of model complexity for all 9 models (including number of parameters, training time, test time, and accuracy for a single run) is presented in Table 5S of the manuscript Supplementary Information.

### Expert biomarker labeling and CNN biomarker presence prediction

When odds ratio values based on Fisher’s Exact Test are greater than 1, they indicate higher risk for the disease outcome. Results of feature presence labeling showed that the top two features with highest odds ratios for CNNs for all (NNV + NV) AMD risk are consistent with those for clinicians (CNV and GA); however, for NV AMD specifically, experts and CNNs only agreed on CNV with highest odds ratios. After that, Scar had second highest odds ratios for experts, while GA had second highest odds ratios for CNNs. This discrepancy could be attributed to the fact that in advanced stages of NV AMD, where a scar has developed, there are often areas of GA and increased light penetrance present alongside areas of scarring. Regarding clinical practice, treatment decisions for NV AMD are predicated on the presence or absence of IRF/SRF and scar, and less so on the presence of GA, which is typically thought of more as a feature of advanced NNV AMD. The fact that the CNN detects GA commonly as a feature of NV AMD demonstrates that this feature is present in a large proportion of NV AMD patients as well. In fact, while experts and AI agree on rank order of all 5 features for any AMD diagnosis, the AI attributes a larger odds ratio than human experts to GA, Scar, and Large PED than to IRF/SRF only for NV AMD diagnosis, potentially because these features, like GA, are more visible on OCT structural information than IRF/SRF (requiring careful manual detection on b-scans). Moreover, as IRF/SRF is a measure of disease activity, this feature is at times present or absent in patients with known NV AMD. When patients are responding well to intravitreal therapies, there is usually a concurrent decrease in IRF/SRF. This fluctuating nature of IRF/SRF would therefore affect the odds ratio calculations for the AI and also explains the variation in IRF/SRF feature presence both for experts and AI for the clinical algorithm proportion analysis discussed in the next paragraph. Fisher’s Exact Test analysis for the 28 false-positive eyes (expert diagnosed non-AMD eyes that were classified as either NNV or NV AMD by model (4)) resulted in odds ratios of near 0 (0.05 with non-significant p values) for all five features. This indicates that false positives were indeed a very small fraction of AI-predicted features; hence there exist no major discrepancies between the AI and experts for truly significant features indicative of (NNV or NV) AMD diagnosis. This finding strengthens the value/trustworthiness of this approach. It is also interesting to note that if the clinical algorithm were used on AI feature predictions as an indicator of overall disease outcome specifically for NV AMD (using AI-predicted presence of IRF/SRF or AI-predicted presence of CNV as a proxy for NV AMD diagnosis), then the AI achieves a sensitivity of 0.729 and a specificity of 0.949, higher than the independent disease classification sensitivities and specificities of models (4) and (9) for the NV AMD class (Table 3). The same procedure is not sufficient for detecting NNV AMD, however, as the clinical algorithm (absence of IRF/SRF and absence of CNV) is not unique to NNV AMD patients but in fact also occurs for non-AMD patients.

Proportion analysis (feature presence or absence for patients in a particular disease class) indicates that the CNN learned the absence of CNV and IRF/SRF for NNV AMD patients better that it learned *presence of these two features* for detecting NV AMD, while presence of these two features is used for the clinical algorithm (shown by the higher percentage of NV AMD patients labeled as positive for these two features by clinicians). So, AI-predicted labels are consistent with the clinical algorithm that expects NV AMD patients to be positive for CNV or IRF/SRF, and NNV AMD patients to be negative for CNV and IRF/SRF, but not to as high of an extent as the expert labels. The fluctuating nature of IRF/SRF in patients responding well to intravitreal therapies, as described in the previous paragraph, may also explain the lower proportion of **IRF/SRF+ **markings within NV AMD patients both for experts and the AI.

### Analysis of false positives and true positives to explain CNN mechanisms

**Figure 4:**
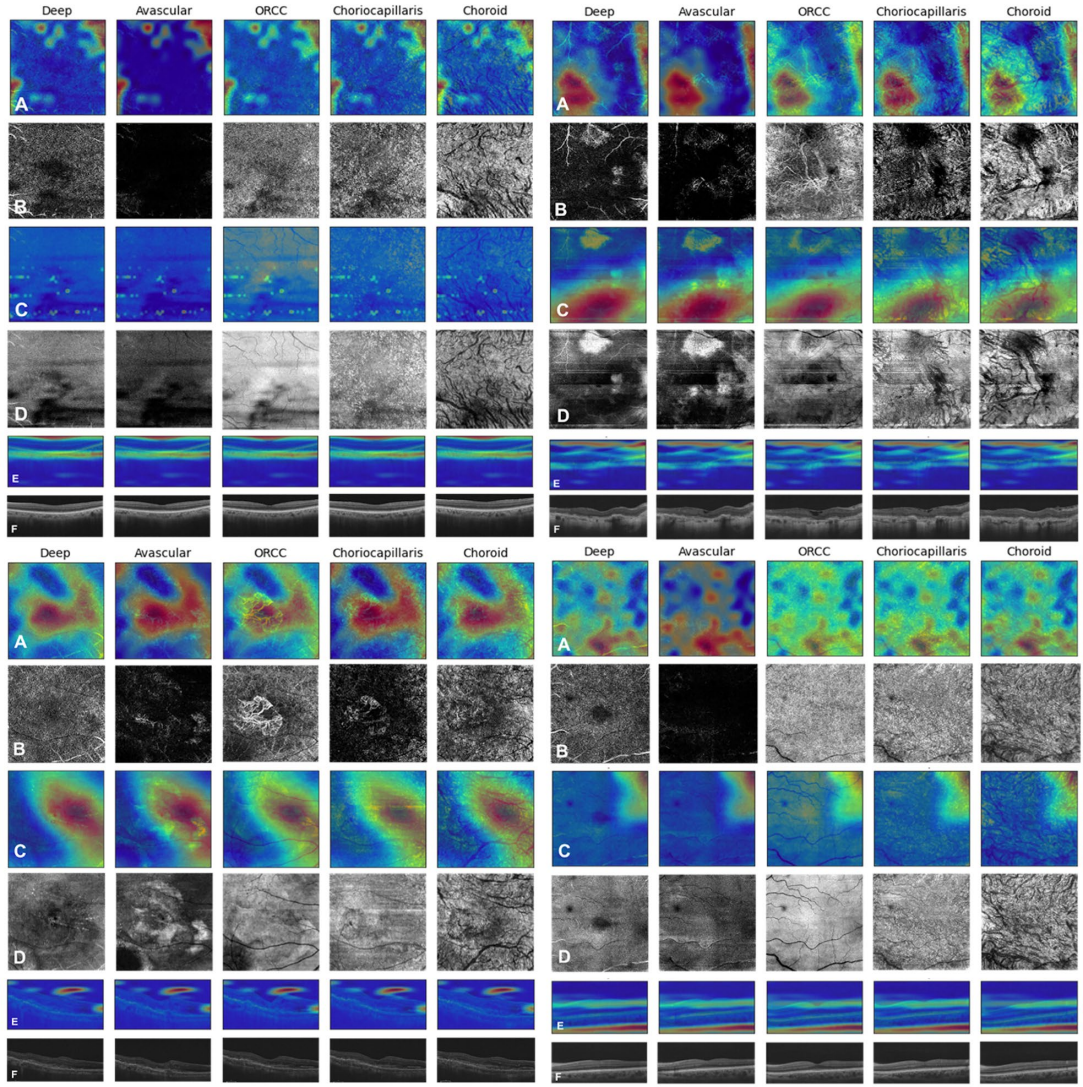
Grad-CAMs of FP (top left), FN (top right), TP (bottom left), and TN (bottom right) example. Rows A, C, and E show Grad-CAMs, while corresponding original OCTA, OCT structure, and HD 5-line b-scan layers are shown in rows B, D, and F. In the FP at top left, bright dots in row C structural Grad-CAMs are likely vitreous shadowing artifacts, misconstrued by the CNN as drusen, suggesting the model has learned the importance of drusen for NNV AMD. An NV AMD patient mis-classified as non-AMD is shown at top right due to misinterpretation of imaging artifacts. In the TP example (bottom left), the model has detected patterns consistent with CNV, shown by the highlighted red/yellow regions in the center of the Grad-CAMs in rows A and C. In the TN at bottom right, the presence of minimal yellow/red highlighted areas, especially in the structural layers, is consistent with a correct classification.

Figure 4 shows Gradient Weighted Class Activations Maps, or Grad-CAMs^24^, of a False Positive (FP, top left), False Negative (FN, top right), True Positive (TP, bottom left), and True Negative (TN, bottom right). Red/yellow regions in the Grad-CAMs indicate image regions that contribute to the CNN’s classification decision. In all cases, rows A, C, and E show Grad-CAMs of OCTA, OCT structural, and HD 5-line b-scan layers, respectively, and rows B, D, and F show corresponding original OCTA, OCT structural, and HD 5-line b-scan images. The FP is a non-AMD subject misclassified as having NNV AMD. Interestingly, the CNN seems to be highlighting areas that correspond to vessels in the OCT structural layers as well as spots that resemble drusen (typically a hallmark of NNV AMD) but are, in fact, high contrast regions caused by shadowing artifacts from vitreous floaters (as shown in the Grad-CAMs of OCT structural layers in row C of Fig. 4 (top left); corresponding original OCT structural images are shown in row D and demonstrate presence of vitreous shadowing artifacts)^18^. Red/yellow areas of Grad-CAM images of the HD 5-line b-scan images of the FP patient (top left, row E) highlight areas of the choroid and retinal pigment epithelium (RPE) inferiorly and the vitreous cavity superiorly instead of the retinal layers. Perhaps the highlighted areas in the choroid/RPE were misinterpreted by the CNN as drusen, thereby classifying the patient as NNV AMD. The FN in Fig. 4 (top right) shows a patient diagnosed with NV AMD classified by the CNN as non-AMD, perhaps due to the red/yellow areas of the Grad-CAM highlighting darker lines resulting from motion artifacts in the middle and lower portions of the structural OCT images, as well as a disciform scar present in the upper portion of the image. Interestingly, the Grad-CAMs of the OCTA (row A) appear to highlight the CNV lesion in the middle-left portion, but the presence of the other artifacts led to the overall misclassification. The red/yellow regions in Grad-CAMs of the HD 5-line b-scans of the FN patient (top right, row E) highlight areas in the vitreous cavity as well as an area of retinal thickening nasally due to underlying retinal scarring and fibrosis from the CNV. It should be noted that the b-scan of this patient with poor central fixation is of lower quality and shows significant areas of atrophy and increased light penetrance through the RPE as well as an epiretinal membrane. This, in addition to the motion artifact in the OCT structural images, could explain why the CNN was not capable of categorizing this patient as NV AMD. The TP in Fig. 4 (bottom left) and TN in Fig. 4 (bottom right) include patients correctly classified as NV AMD and as non-AMD, respectively. In the TP (bottom left), the CNN appears to recognize presence of emerging CNV patterns; Grad-CAMs of b-scans of the TP patient (bottom left, row E) highlight areas in the vitreous cavity that lie superior to a region of thickened retina and distorted retinal contour due to the presence of IRF from an underlying CNV. An area of atrophy, loss of ellipsoid zone, and minimal SRF are also highlighted nasally. These areas in the b-scan, in addition to the clearly highlighted CNV membrane in the OCTA Grad-CAMs (row A) of the ORCC layer, demonstrate that the CNN was capable of recognizing true signs of NV AMD and categorized the patient correctly as such. Grad-CAMs of b-scans of the TN patient (bottom right, row E) show that the CNN is correctly highlighting areas corresponding to retinal layers. Additionally, an area external to the choroid is highlighted by the CNN. After closely reviewing the b-scans for this patient, it is not clear to us why this area which corresponds to the sclera is highlighted; it appears that the contrast between the vascular choroid and the avascular sclera has captured the CNN’s attention. For all of these examples, the Grad-CAM is examining the final convolutional layer of each input image modality network prior to feature concatenation, using model (4).

## Conclusions and future directions

All models described here achieved above-chance multi-class performance at detection of Non-AMD vs. early and late stages of NNV AMD vs. NV AMD, suggesting promise for translating our approach to the clinic. By leveraging existing strengths of state-of-the-art CNN architectures, such as feature concatenation via long skip connections, our study paves the way for optimization of our top-performing CNN architectures [models (4) and (9)] for the best input modalities discovered here (combining OCTA, OCT structure, and b-scans). Model (4), combining OCTA, OCT structure, and HD 5-line b-scans and model (9), combining OCTA, OCT structure, HD 5-line b-scans, and 2D b-scan flow images exhibited best performance compared to single and dual modality combinations of OCTA, OCT structure, and b-scan information. Current limitations in the form of overfitting of our models could potentially be improved by using an asymmetric loss function^25^, customized for handling class imbalance by more heavily weighting samples of a class present in lower quantity. Adding residual blocks or replacing conventional convolutional kernels with spatial or depth-wise separable kernels may help improve accuracy^26,27^. Attempting to leverage knowledge gained from low-resolution 2D b-scan flow images to inform utility of HD 5-line b-scan cubes (or vice versa) may further help to reduce the parameter space and thus prevent overfitting, by enhancing emphasis on the most critical layers/images. Salvi and colleagues^28^ describe a number of pre-processing as well as post-processing techniques (including de-speckling) that help improve deep learning model performance. Although speckle noise is typically the predominant source of noise in OCT, we did not observe speckle noise as a significant issue in our data; we anticipate that incorporation of pre-processing approaches such as data augmentation may potentially improve model performance in future iterations of this work. Among biomarkers included in our labeling analysis, experts and CNNs agree on CNV and GA as the top two with highest odds for any (NNV + NV) AMD diagnosis when present and agree on CNV presence as indicating high odds of NV AMD diagnosis, helping to build trust/credibility in such AI technology. Consistency between experts and CNNs for features predictive of NV AMD may be enhanced by segmentation/localization of image biomarkers within b-scans beyond simply detection of biomarker presence. Using CNN feature predictions in tandem with the clinical algorithm enabled improvement in sensitivity and specificity of NV AMD detection, compared to CNN image-level disease classification. Grad-CAMs serve a valuable role to further explain CNN mechanism of function, as they showed that CNNs are detecting CNV presence in true positive OCTA, structural, and b-scan images. Detection of late-stage AMD and presence of AMD biomarkers from OCTA/OCT structural images and b-scans via CNNs has tremendous potential for translation to the clinic to expedite workflow for screening of early and late-stage AMD patients.

## Supplementary Information

Supplementary Information.

## Data Availability

The code developed in this study can be made available upon request.

## Data availability

The data collected in this study can be made available upon request.
