# Supplementary material for: A multimodal deep learning system to distinguish late stages of AMD and to compare expert vs. AI ocular biomarkers
Source: Sci Rep. 2022 Feb 16;12:2585. doi: 10.1038/s41598-022-06273-w (PMC8850456; doi:10.1038/s41598-022-06273-w)
Supplement: Supplementary file 1 — Supplementary Information. [file 41598_2022_6273_MOESM1_ESM.pdf]

Supplementary Information: A Multimodal Deep Learning System to Distinguish Late Stages of AMD and to Compare Expert vs. AI Ocular Biomarkers

Kaveri A. Thakoor[1,*], Jiaang Yao[2], Darius Bordbar[3], Omar Moussa[3], Weijie Lin[3], Paul Sajda[1,2,4], Royce W. S. Chen[3]

1. Columbia University, Department of Biomedical Engineering, New York, 10027, USA
2. Columbia University, Department of Electrical Engineering, New York, 10027, USA
3. Edward S. Harkness Eye Institute, Columbia University Irving Medical Center, Department of Ophthalmology, New York, 10032, USA
4. Columbia University, Department of Radiology (Physics), New York, 10027, USA

*k.thakoor@columbia.edu

Confusion Matrices and Test Accuracies of All 9 Model Architectures

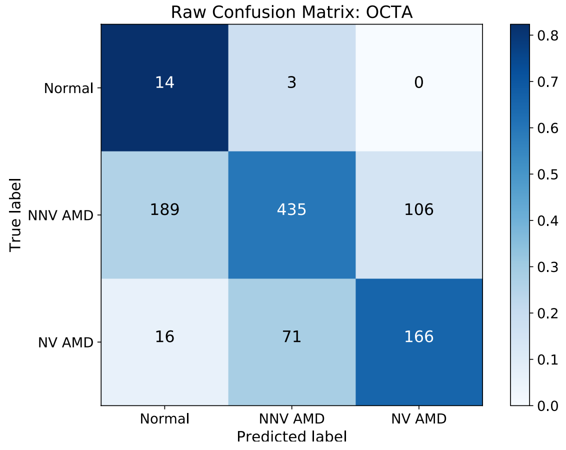

Model 1 Test Accuracy: 61.5%

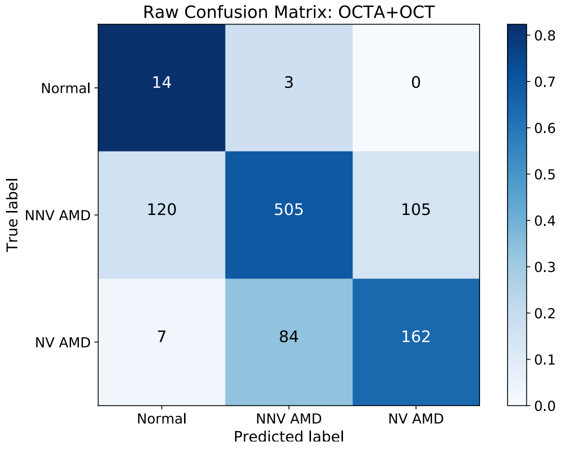

Model 2 Test Accuracy: 68.1%

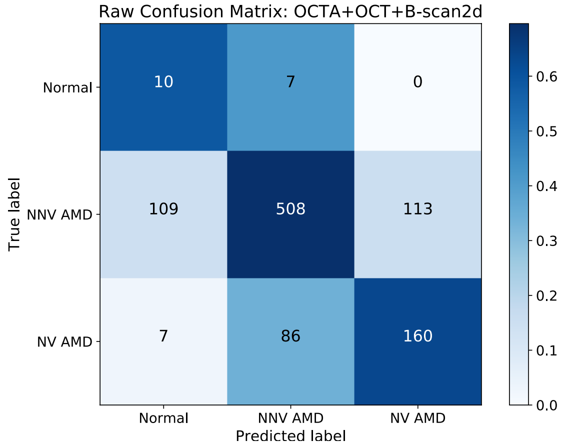

Model 3 Test Accuracy: 67.8%

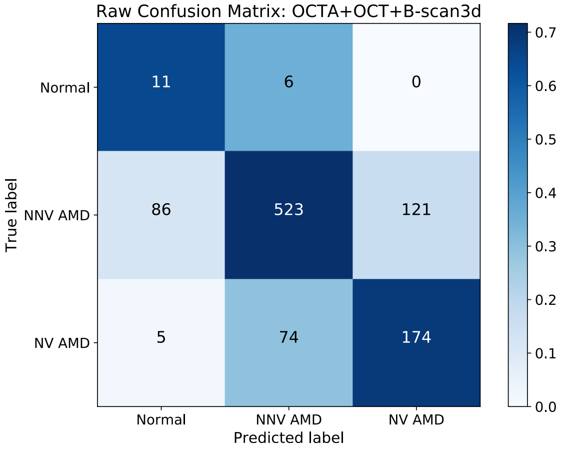

Model 4 Test Accuracy: 70.8%

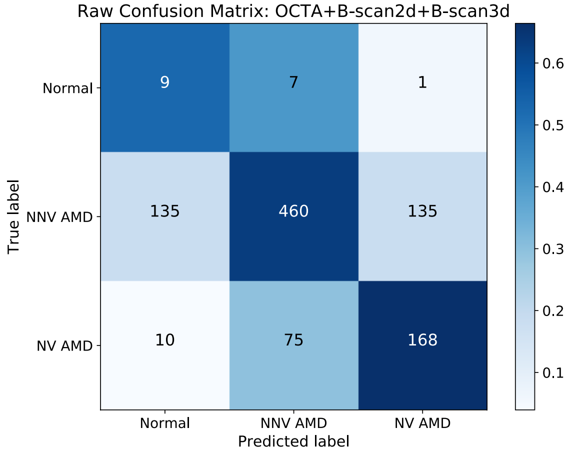

Model 5 Test Accuracy: 63.7%

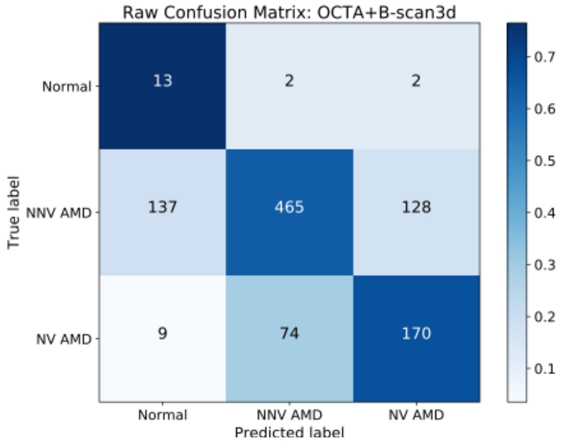

Model 6 Test Accuracy: 64.8%

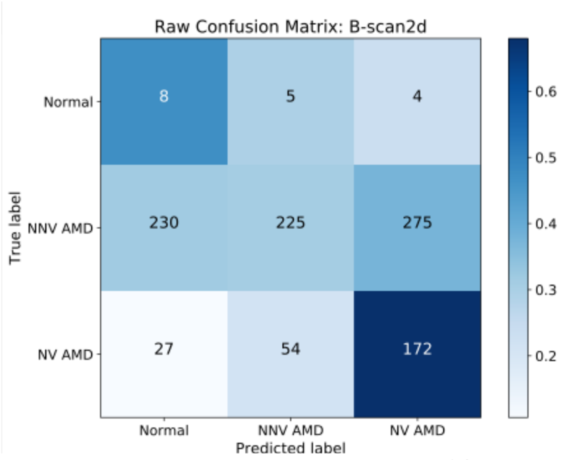

Model 7 Test Accuracy: 40.5%

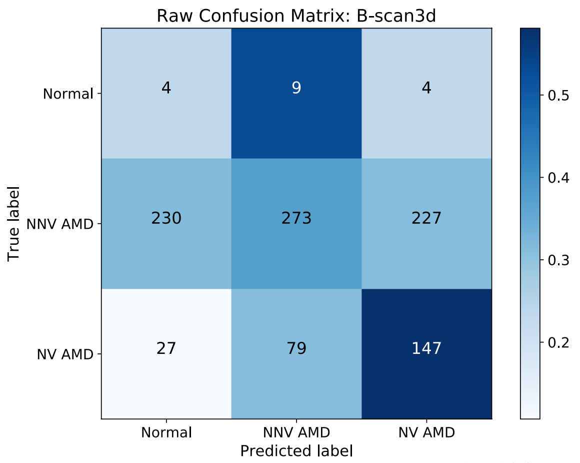

Model 8 Test Accuracy: 42.4%

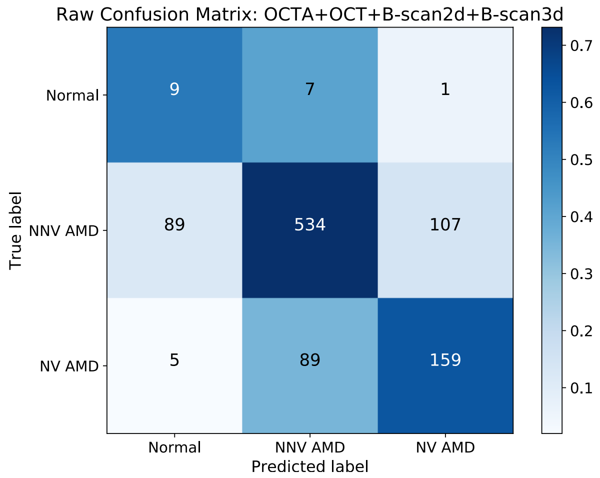

Model 9 Test Accuracy: 70.2%

Table 1S: Odds Ratios for experts and for AI for five biomarkers most indicative of NNV or NV AMD are shown in the left half of the table. For all AMD, both experts and AI agree on rank order, with CNV and GA at the top. Experts and AI odds ratios for five biomarkers specifically for NV AMD are shown in the right half of the table. For NV AMD, beyond CNV at the top, experts and AI show some variation in rank order. Features exhibiting agreement in rank between experts and AI are bolded. These results were generated using Model (9) and 482 of 501 eyes.

All AMD (NV or NNV AMD)			***NV AMD Only***		
Feature	***Human Expert*** ***Rank (Odds Ratio)***	***Algorithm*** ***Rank (Odds Ratio)***	***Feature***	***Human Expert*** ***Rank (Odds Ratio)***	***Algorithm*** ***Rank (Odds Ratio)***
**CNV**	**1 (39)**	**1 (38)**	**CNV**	**1 (4304)**	**1 (436)**

**GA**	**2 (34)**	**2 (30)**	Scar	2 (124)	3 (94)
Scar	**3 (21)**	**3 (18)**	IRF/SRF	3 (103)	4 (65)
Large PED	**4 (18)**	**3 (18)**	GA	4 (99)	2 (113)
IRF/SRF	**4 (18)**	**4 (14)**	Large PED	5 (60)	5 (62)

Table 2S: Proportion of patients diagnosed with NV AMD and NNV AMD (by experts and the AI) labeled for presence (+) or absence (-) of CNV and IRF/SRF biomarkers by the experts and the AI, respectively using Model (9) and 482 of 501 eyes.

**Feature/Biomarker and Labeler**	**NV AMD (Feature+)**	**NNV AMD (Feature-)**
CNV by Expert	100%	99%
CNV by AI	81%	94%
IRF/SRF by Expert	56%	99%
IRF/SRF by AI	37%	99%

Table 3S: Odds Ratios for experts and for AI for five biomarkers most indicative of NNV or NV AMD are shown in the left half of the table. For all AMD, both experts and AI agree on rank order, with CNV and GA at the top. Experts and AI odds ratios for five biomarkers specifically for NV AMD are shown in the right half of the table. For NV AMD, beyond CNV at the top, experts and AI show some variation in rank order. Features exhibiting agreement in rank between experts and AI are bolded. These results were generated using Model (2) (without b-scans) and only 346 of 501 eyes.

All AMD (NV or NNV AMD)			***NV AMD Only***		
Feature	***Human Expert*** *Rank (Odds Ratio)*	***Algorithm*** *Rank (Odds Ratio)*	***Feature***	***Human Expert*** *Rank (Odds Ratio)*	***Algorithm*** *Rank (Odds Ratio)*
**CNV**	**1 (31)**	**1 (34)**	**CNV**	**1 (2480)**	**1 (453)**
**GA**	**2 (23)**	**2 (12)**	IRF/SRF	2 (130)	3 (43)

Large PED	**3 (18)**	**2 (12)**	Large PED	3 (95)	2 (60)
IRF/SRF	**4 (17)**	**3 (9)**	Scar	4 (81)	3 (43)
Scar	**5 (14)**	**3 (9)**	GA	5 (60)	4 (40)

Table 4S: Proportion of patients diagnosed with NV AMD and NNV AMD (by experts and the AI) labeled for presence (+) or absence (-) of CNV and IRF/SRF biomarkers by the experts and the AI, respectively using Model (2) (without b-scans) and 346 of 501 eyes.

Feature/Biomarker and Labeler	NV AMD (Feature+)	NNV AMD (Feature-)
CNV by Expert	98%	100%
CNV by AI	86%	92%
IRF/SRF by Expert	63%	100%
IRF/SRF by AI	35%	99%

Table 5S: Number of parameters, training time, test time, and test accuracy for a single run of each of the 9 models developed and analyzed in this study. Values for top-performing models (4) and (9) are bolded.

Model Name	Number of parameters	Training time (s)	Test time (s)	Test Accuracy
**Model 1**	121,278	245.646	0.834	0.500
**Model 2**	263,801	449.538	1.363	0.604
**Model 3**	474,474	504.025	1.414	0.625
**Model 4**	**412,724**	**544.235**	**1.974**	**0.646**
**Model 5**	480,874	504.688	1.500	0.563
**Model 6**	559,641	350.433	1.198	0.469
**Model 7**	211,828	14.854	0.384	0.333
**Model 8**	439,518	72.612	0.674	0.469
**Model 9**	**623,397**	**462.901**	**2.026**	**0.656**
